# Are Women Who Work in Bars, Guesthouses and Similar Facilities a Suitable Study Population for Vaginal Microbicide Trials in Africa?

**DOI:** 10.1371/journal.pone.0010661

**Published:** 2010-05-14

**Authors:** Andrew Vallely, Ian R. Hambleton, Stella Kasindi, Louise Knight, Suzanna C. Francis, Tobias Chirwa, Dean Everett, Charles Shagi, Claire Cook, Celia Barberousse, Deborah Watson-Jones, John Changalucha, David Ross, Richard J. Hayes, for the Microbicides Development Programme

**Affiliations:** 1 London School of Hygiene and Tropical Medicine, London, United Kingdom; 2 African Medical and Research Foundation, Mwanza, Tanzania; 3 National Institute for Medical Research, Mwanza, Tanzania; 4 Tropical Medicine Research Institute, The University of the West Indies, Bridgetown, Barbados, West Indies; University of Cape Town, South Africa

## Abstract

**Background:**

A feasibility study was conducted to investigate whether an occupational at-risk cohort of women in Mwanza, Tanzania are a suitable study population for future phase III vaginal microbicide trials.

**Methodology/Principal Findings:**

1573 women aged 16–54 y working in traditional and modern bars, restaurants, hotels, guesthouses or as local food-handlers were enrolled at community-based reproductive health clinics, provided specimens for HIV/STI and pregnancy testing, and asked to attend three-monthly clinical follow-up visits for 12-months. HIV positive and negative women were eligible to enter the feasibility study and to receive free reproductive health services at any time. HIV prevalence at baseline was 26.5% (417/1573). HIV incidence among 1156 sero-negative women attending at baseline was 2.9/100PYs. Among 1020 HIV sero-negative, non-pregnant women, HIV incidence was 2.0/100PYs, HSV-2 incidence 12.7/100PYs and pregnancy rate 17.8/100PYs. Retention at three-months was 76.3% (778/1020). Among 771 HIV sero-negative, non-pregnant women attending at three-months, subsequent follow-up at 6, 9 and 12-months was 83.7%, 79.6%, and 72.1% respectively. Older women, those who had not moved home or changed their place of work in the last year, and women working in traditional bars or as local food handlers had the highest re-attendance.

**Conclusions/Significance:**

Women working in food outlets and recreational facilities in Tanzania and other parts of Africa may be a suitable study population for microbicide and other HIV prevention trials. Effective locally-appropriate strategies to address high pregnancy rates and early losses to follow-up are essential to minimise risk to clinical trials in these settings.

## Introduction

In many developed and developing countries, HIV prevention trials are usually undertaken among vulnerable, disadvantaged communities at high-risk of HIV and STIs, where HIV incidence rates make randomised controlled clinical trials feasible[1], [2] but where poverty and social exclusion mean ethical considerations are paramount[3], [4]. Such populations may include female sex workers, injecting drug users, men who have sex with men, prison inmates and the homeless[5], [6], [7], [8], [9], [10], [11], [12], [13], [14].

There are considerable ethical and logistical challenges to consider when planning HIV prevention trials in such populations, for example, the need to develop effective locally-appropriate informed consent procedures[4], [15], [16], [17], [18], [19], of ensuring that trial participation does not exacerbate stigma, vulnerability or social harm[20], [21], [22]; and to ensure that appropriate locally-agreed standards of clinical care are made available to trial participants, particularly those who seroconvert during the trial or who are HIV positive at screening[23], [24], [25].

Recruiting and retaining participants from high-risk cohorts is often problematic. In many settings it is difficult to access and meaningfully engage with vulnerable groups in the community[11], [12]. Providing accurate information about trial objectives and procedures; dispelling fears around disclosure and confidentiality; and reducing distrust and suspicion between potential participants and external researchers may therefore be difficult, and may hinder trial enrolment. High mobility is common in many vulnerable groups, which makes tracing those who default from follow-up difficult, necessitating the use of community fieldworkers or peer educators[26]. Volunteers from vulnerable groups may be reluctant to attend established health services due to travel and opportunity costs and the way in which they are treated by health centre staff so that it is often necessary to establish community-based study clinics, which may need to operate at unusual hours or at weekends in order to encourage potential participants to attend[4], [12], [26]. In addition to these considerations, researchers planning vaginal microbicide trials also need to take into account pregnancy rates and the prevalence and nature of intravaginal hygiene practices in any proposed study population.

The search for female-controlled methods of HIV prevention has led to a concerted international effort to develop vaginal microbicides: topical chemical agents, which when administered vaginally prior to sexual intercourse would prevent HIV and ideally other sexually transmitted infections (STIs)[27], [28]. Mwanza is one of six centres in Sub-Saharan Africa participating in the Microbicides Development Programme (MDP), an international partnership for the development of vaginal microbicides for HIV prevention, funded by the UK Department for International Development and Medical Research Council (MRC)[29].

In preparation for the MDP301 randomised placebo-controlled efficacy and safety trial of the candidate vaginal microbicide PRO2000/5 Gel (Indevus Pharmaceuticals, USA), we carried out a feasibility study in an occupational cohort of women at high-risk of HIV/STIs in ten administrative wards in Mwanza City, northern Tanzania between July 2002 and December 2004. Women working in food and recreational facilities, including modern bars, traditional bars (*vilabu* or pombe shops), restaurants, hotels, guesthouses, groceries and as informal food vendors (*mamalishe*), were eligible to participate. In Tanzania, high rates of HIV and STIs have been reported among women working in such facilities in areas situated along major transit routes[30], [31], [32], [33] and in commercial centres adjacent to newly-established goldmines[34], [35]. Some women in this occupational group are reported to periodically supplement their income through transactional sex[35], [36] and, although not necessarily perceived as commercial sex workers within the wider community, are nonetheless at increased risk of STIs and HIV infection[31], [33], [34].

The objectives of the feasibility study were to: test the feasibility of recruiting and retaining sufficient numbers of women for a subsequent microbicides trial; assess baseline HIV and STI prevalence, HIV incidence and pregnancy rates; investigate methods for improving the reliability and validity of reported sexual behaviour data including condom use and intravaginal practices; and investigate the acceptability of vaginal microbicide gels among women and their sexual partners.

The baseline socio-demographic and behavioural characteristics of 1573 participants enrolled in the feasibility study and the factors associated with loss to follow-up at three-months have previously been described[37]. In this paper, we present data on cohort retention, factors associated with loss to follow-up, and HIV, STI and pregnancy incidence over 12 months of follow-up among initially HIV sero-negative, non-pregnant women who would be eligible for future phase III HIV prevention trials. We use the findings to examine whether this is a suitable study population in which to conduct such clinical trials in Africa.

## Methods

### Study design

The design of the study, study population and laboratory methods have been described previously[37]. Briefly, following participatory community mobilisation and mapping activities, weekly community-based reproductive health clinics were established in selected guesthouses in ten administrative wards in Mwanza City, northern Tanzania by October 2002. In order to avoid exacerbating stigma, social exclusion or harm as a result of study participation and to investigate the feasibility of recruiting women for a variety of future HIV-related clinical research activities both HIV positive and negative women were eligible to participate in the Mwanza feasibility study. The study design did not therefore include a preliminary screening assessment (e.g. to confirm negative HIV and pregnancy status) and participants were directly enrolled at their first clinic visit.

### Study procedures and investigations

Free reproductive health services, including syndromic management of STIs, family planning, health education, and voluntary HIV counselling and testing (VCT) were provided. Participants were asked to attend a study clinic every three months, when a study nurse collected demographic and sexual behaviour information using a pre-tested questionnaire in Swahili. A gynaecological examination was offered at entry and six monthly clinical follow-up visits when vaginal swabs for bacterial vaginosis and *Trichomonas vaginalis*, cervical swabs for *Neisseria gonorrhoeae* and *Chlamydia trachomatis*, urine for pregnancy testing and venous blood for HIV, syphilis and *Herpes simplex* type-2 (HSV-2) serology were also collected. Women with symptoms and clinical signs of STIs were managed according to Tanzanian national syndromic management guidelines[38]. Women were encouraged to use the free drop-in reproductive health service at any time between booked appointments.

### Ethics

Ethical clearance was obtained from the National Medical Research Coordinating Committee in Tanzania and the Ethics Committee of the London School of Hygiene and Tropical Medicine, UK. Written informed consent (signature or witnessed thumbprint) was obtained from all participants prior to enrolment.

### Statistical Methods

**Figure 1:**
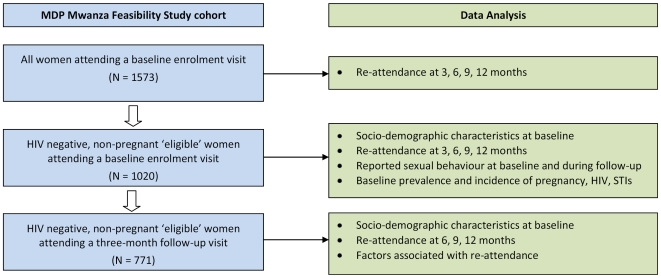
Data analysis summary by sub-cohort, MDP Mwanza Feasibility Study.

Data were double-entered and verified. We calculated attendance rates at three-monthly visits for three distinct groups: the complete cohort of HIV positive and negative women attending a baseline clinic visit (N = 1573); all HIV-negative, non-pregnant women from this complete baseline cohort (N = 1020); and all HIV-negative, non-pregnant women who attended a first follow-up visit at three-months (N = 771) (Figure 1). The sub-cohorts of 1020 and 771 subjects represent women who would be considered broadly eligible for entry into a phase III vaginal microbicide trial such as MDP301. Due to the lack of a screening visit in the Mwanza feasibility study design we considered that retention rates among the sub-cohort of 771 ‘eligible’ women who re-attended for the first follow-up visit were more likely to be predictive of future retention in a phase III clinical trial in this setting than in the larger sub-cohort of 1020 women or the complete cohort of 1573 subjects.

We present selected characteristics of ‘eligible’ women, stratified by the type of facility in which they are employed (*mamalishe* or pombe shop, guesthouse or hotel, restaurant or bar or grocery). We calculated the baseline prevalence and incidence of pregnancy and serological markers including HIV; and the prevalence of STIs and aspects of sexual behaviour at each follow-up visit. We tested for trends in prevalence over time by fitting a longitudinal logistic regression model for each STI and sexual behaviour outcome, which allowed for correlations induced by repeat visits of each participant. We included time in each model by including visit attendance (baseline then three-monthly follow-up over 12-months), and assessed its effect using a likelihood ratio test.

To examine factors contributing to cohort re-attendance, we created a new variable for the number of follow-up visits attended after the three-month clinic visit (‘attendance’). Each participant could therefore miss one, two, or three subsequent visits. We treated this outcome variable as ordinal and used ordinal logistic regression to examine factors associated with re-attendance, so that an odds ratio >1.0 was associated with a better attendance profile. Models were adjusted for age, clinic site, facility type, and other variables that were statistically significant (P<0.05). For this regression analysis we constructed two composite variables with three levels (low, moderate and high): ‘travel’ based on time away from home, and ‘permanence’ based on moving home or job in the previous year. We used Stata for all analyses (Version 10, StataCorp LP, College Station, Texas, US).

## Results

### General characteristics of the study population

Participatory community mapping conducted in March 2002 showed that there were around 2494 women working in 953 food and recreational facilities in ten administrative wards in Mwanza City[37]. Of 1573 women attending a first clinic visit, 1020 (64.8%) were HIV sero-negative, had a negative urine pregnancy test and would have been considered broadly eligible for enrolment into a future vaginal microbicide trial (Table 1). Around 24% (242/1020) of women in this sub-population failed to re-attend a second clinic visit at three-months; subsequent retention was also poor in this group. Of the 778 women who re-attended at three-months, 771 would have been considered eligible for enrolment into a future microbicide trial based on the above criteria. Re-attendance in this sub-population at subsequent three-monthly clinical follow-up visits was 83.7%, 79.6% and 72.1%.

**Table 1:** Re-attendance among women eligible to enrol in a future microbicide trial.

	Attendance		
Visit	Full cohort	Eligible and attending at baseline 1	Eligible and attending at 3 mo. 2
Baseline	1573	1020	
3 month	1165 (74.1%)	778 (76.3%)	771
6 month	1079 (68.6%)	726 (71.2%)	645 (83.7%)
9 month	1006 (64.0%)	680 (66.7%)	614 (79.6%)
12 month	905 (57.5%)	607 (59.5%)	556 (72.1%)
**Overall attendance rate at 12-months (95% CI)** 3	**66.0% (64.9–67.2)**	**68.4% (67.0–69.8)**	**78.5% (76.7–80.1)**

**1** At baseline, there were 1573 attendees. Of these 1156 (73.5%) were HIV negative, 1409 (89.6%) were not pregnant, and 1020 (64.8%) were both HIV negative and not-pregnant and therefore met broad eligibility criteria for enrolment into a future microbicide trial.

**2** At the 3-month clinic visit, 778/1020 (76.3%) women originally considered eligible for enrolment re-attended; 7/778 women sero-converted between baseline and 3 mo. visits leaving a total of 771 ‘eligible’ women at 3-months.

**3** Attendance  =  % of all possible follow-up visits that were actually attended.

The socio-demographic characteristics of the 1020 eligible women attending at baseline and the 771 eligible women attending at three-months were broadly similar in terms of facility type, level of education, literacy and ethnicity (Table 2) and reflected the distribution observed in the full feasibility study cohort of 1573 women[37]. Comparing the 242 women who did not attend at three-months with the 778 women who did, women who did not re-attend tended to be younger (difference in mean age 4.9 y; p<0.001); were less likely to be married (p<0.001); had travelled more frequently in the three-months before recruitment (difference in travel scores p = 0.01); and were more mobile in terms of recent changes in accommodation and place of work (difference in permanence scores p<0.001).

**Table 2:** Selected socio-demographic characteristics of eligible sub-cohorts at baseline (n = 1020) and 3 mo. visit (n = 771).

	Baseline (n = 1020)				3-months (n = 771)			
	Mamalishe/Pombe (n = 654; 64.1%)	Guesthouse/Hotel (n = 164; 16.1%)	Restaurant/Bar/Grocery (n = 202; 19.8%)	All (N = 1020)	Mamalishe/Pombe (n = 517; 67.1%)	Guesthouse/Hotel (n = 115; 14.9%)	Restaurant/Bar/Grocery (n = 139; 18.0%)	All (N = 771)
**Age (mean, SD)**	30.8 (8.7)	27.1 (6.5)	27.1 (7.0)	29.5 (8.2)	31.9 (8.7)	28.2 (6.8)	28.0 (7.0)	30.6 (8.4)
**Age (years)** 1								
<20	49 (7.5%)	16 (9.8%)	20 (9.9%)	85 (8.3%)	25 (4.8%)	6 (5.2%)	10 (7.2%)	41 (5.3%)
20–24	124 (19.0%)	50 (30.5%)	71 (35.2%)	245 (24.0%)	89 (17.3%)	34 (29.6%)	41 (29.5%)	164 (21.3%)
25–34	274 (42.0%)	76 (46.3%)	86 (42.6%)	436 (42.8%)	213 (41.3%)	54 (47.0%)	69 (49.6%)	336 (43.6%)
35+	206 (31.6%)	22 (13.4%)	25 (12.4%)	253 (24.8%)	189 (36.6%)	21 (18.3%)	19 (13.7%)	229 (29.7%)
**Education**								
Primary incomplete	184 (28.1%)	22 (13.4%)	37 (18.3%)	243 (23.8%)	144 (27.9%)	16 (13.9%)	25 (18.0%)	185 (24.0%)
Primary complete	422 (64.5%)	94 (57.3%)	142 (70.3%)	658 (64.5%)	336 (65.0%)	63 (54.8%)	94 (67.6%)	493 (63.9%)
Secondary	30 (4.6%)	43 (26.2%)	19 (9.4%)	92 (9.0%)	21 (4.1%)	33 (28.7%)	16 (11.5%)	70 (9.1%)
Other	18 (2.8%)	5 (3.1%)	4 (2.0%)	27 (2.7%)	16 (3.1%)	3 (2.6%)	4 (2.9%)	23 (3.0%)
**Marital status**								
Married	342 (52.3%)	23 (14.0%)	38 (18.8%)	403 (39.5%)	282 (54.6%)	16 (13.9%)	26 (18.7%)	324 (42.0%)
Separated/divorced/widowed	218 (33.3%)	78 (47.6%)	97 (48.0%)	393 (38.5%)	177 (34.2%)	55 (47.8%)	67 (48.2%)	299 (38.8%)
Single	94 (14.4%)	63 (38.4%)	67 (33.2%)	224 (22.0%)	58 (11.2%)	44 (38.3%)	46 (33.1%)	148 (19.2%)
**Ethnic group**								
Sukuma	231 (35.3%)	63 (38.4%)	63 (31.2%)	357 (35.0%)	185 (35.8%)	41 (35.7%)	43 (30.9%)	269 (34.9%)
Other	423 (64.7%)	101 (61.6%)	139 (68.8%)	663 (65.0%)	332 (64.2%)	74 (64.4%)	96 (69.1%)	502 (65.1%)
**Literacy**								
No	124 (19.0%)	16 (9.8%)	25 (12.4%)	165 (16.2%)	101 (19.5%)	12 (10.4%)	14 (10.1%)	127 (16.5%)
Yes	530 (81.0%)	148 (90.2%)	177 (87.6%)	855 (83.8%)	416 (80.5%)	103 (89.6%)	125 (89.9%)	644 (83.5%)
**Travel** 2								
Low	488 (74.6%)	88 (53.7%)	114 (57.0%)	690 (67.8%)	383 (74.1%)	68 (59.1%)	76 (55.1%)	527 (68.4%)
Moderate	146 (22.3%)	60 (36.6%)	63 (31.5%)	269 (26.4%)	121 (23.4%)	39 (33.9%)	48 (34.8%)	208 (27.0%)
High	20 (3.1%)	16 (9.8%)	23 (11.5%)	59 (5.8%)	13 (2.5%)	8 (7.0%)	14 (10.1%)	35 (4.6%)
**Permanence** 3								
High	315 (48.2%)	45 (27.6%)	45 (22.3%)	405 (39.8%)	269 (52.1%)	34 (29.8%)	39 (28.1%)	342 (44.5%)
Moderate	165 (25.3%)	51 (31.3%)	63 (31.2%)	279 (27.4%)	127 (24.6%)	35 (30.7%)	44 (31.7%)	206 (26.8%)
Low	173 (26.5%)	67 (41.1%)	94 (46.5%)	334 (32.8%)	120 (23.3%)	45 (39.5%)	56 (40.3%)	221 (28.7%)

**1** One participant with no recorded age;

**2** Travel in the three months preceding baseline clinic visit was categorised as: Low (zero nights away from home), Moderate (≥1 night but less than one continuous week away from home) and High (≥1 continuous week away from home on one or more occasions);

**3** Permanence of home and workplace location was categorised as: High (lived in the same house and worked at the same facility during the past year); Moderate (lived or worked at the same location for <1 year and moved house no more than once in the previous year); Low (lived or worked at the same location for <1 year and moved house twice or more in the previous year).

### Prevalence and incidence of pregnancy, HIV and STI

Baseline HIV and STI prevalences have been reported elsewhere[37] but in brief, among the 1573 women who attended a first clinic visit, the prevalence of HIV was 26.5%; pregnancy 9.6%; HSV-2 infection 74.1%; active syphilis (TPPA+/RPR+) 10.1%, bacterial vaginosis 50.7%; gonorrhoea 5.3%; chlamydia 5.7% and trichomoniasis 12.3%. HIV prevalence at baseline increased with age, was lower among local food-handlers (*mamalishe*) and women working in traditional bars, married women and those with fewer sexual partners, and was associated with HSV-2 infection and bacterial vaginosis.

The incidence of HIV among all women who were sero-negative at baseline (1156/1573) was 2.9 per 100 person-years (PYs) (95%CI:1.9–4.3). HIV incidence among *mamalishe*/pombe shop workers (2.0/100PYs [95%CI:1.1–3.6]) and guesthouse/hotel workers (3.5/100PYs [95%CI:1.3–9.3]) was lower than among restaurant/bar workers (5.7/100PYs [95%CI:2.9–11.4]). HIV incidence was highest in those aged 25–34years (4.3/100PYs [95%CI:2.6–7.1]) among whom 15 of the 23 (65%) sero-conversions observed in this sub-population occurred (data not shown).

In the sub-cohort of 1020 eligible (HIV sero-negative, non-pregnant) women who attended at baseline the incidence of HIV was 2.0/100PYs (Table 3). HIV incidence among *mamalishe*/pombe shop workers (1.6/100PYs) and guesthouse/hotel workers (1.0/100PYs) was again lower than among restaurant/bar workers (4.1/100PYs). The incidence of pregnancy in this sub-cohort was 17.8/100PYs and HSV-2 incidence 12.7/100PYs.

**Table 3:** Prevalence and incidence of pregnancy, HIV and STIs among eligible women attending at baseline (n = 1020).

Baseline (n = 1020)						
Pregnancy and serological markers	Prevalent cases	Participants tested	Baseline Prevalence	New Cases	Person years (PYs)	incidence rate per 100 PYs (95% CI) 1
**Pregnancy**	0	1020	**-** 2	111	623.8	17.8 (14.8–21.4)
**Serology**						
HIV	0	1020	**-** 2	14	713.8	2.0 (1.2–3.3)
HSV-2	703	1019	69.0%	19	150.2	12.7 (8.1–19.8)
TPPA+/RPR+^3^	98	1018	9.6%	14	598.0	2.3 (1.4–4.0)
**HIV incidence by facility type**						
Mamalishe/pombe	0	654	**-** 2	8	493.5	1.6 (0.8–3.2)
Guesthouse/hotel	0	164	**-** 2	1	96.1	1.0 (0.1–7.4)
Restaurant/bar/grocery	0	202	**-** 2	5	123.3	4.1 (1.7–9.7)

**1.** Participants were tested for each incident endpoint at baseline, and at three-monthly intervals during follow-up. Incidence was calculated as the number of new cases divided by the total time in years that participants remained in the study without the incident endpoint of interest. Following standard practice, participants were considered censored at their last recorded study visit.

**2.** Pregnancy and HIV seropositive status were initial study exclusion criteria. By definition, HIV prevalence was therefore zero among 1020 eligible women at baseline.

**3.** Incident cases were defined as those who tested positive for both TPPA and RPR for the first time during follow-up and exclude women with active syphilis (TPPA+ and RPR+) at baseline.

The prevalence of gonorrhoea, chlamydia and bacterial vaginosis was significantly lower at 6 and 12-months compared to baseline (Table 4). The prevalence of trichomoniasis also fell from 12.3% at baseline to 6.5% at 12-months (p = 0.09).

**Table 4:** Prevalence of STIs, pregnancy, contraception and reported sexual behaviour at baseline and scheduled follow-up visits among 1020 potential trial participants.

	Baseline (n = 1020)	3 mo. (n = 778)	6 mo. (n = 726)	9 mo. (n = 680)	12 mo. (n = 607)	Odds Ratio/P-value 1
**Genital swabs**						
* N gonorrhoeae* (NG)	5.3% (52/986)	-	1.7% (11/631)	-	1.7% (7/433)	0.66 (0.54–0.82) p<0.001
* C trachomatis* (CT)	5.5% (54/986)	-	1.3% (8/631)	-	3.0% (13/433)	0.75 (0.62–0.90) p = 0.001
* T vaginalis* 2	12.3% (27/220)	-	-	-	6.5% (7/107)	0.81 (0.62–1.05) p = 0.09
Bacterial vaginosis	48.1% (485/987)	-	38.4% (246/641)	-	-	0.75 (0.66–0.84) p<0.001
**Pregnancy**						1.09 (0.72–1.64) p = 0.69
Yes	**-** 3	-	10.0%	-	9.3%	
**Contraceptive prevalence**						1.26 (1.17–1.36) p<0.001
Yes (all methods)	33.9%	34.3%	35.2%	37.5%	43.3%	
**Contraceptive methods**						
Condom	6.3%	7.4%	8.4%	5.9%	9.3%	1.19 (1.05–1.35) p = 0.005
Pill/injection/IUCD	23.4%	20.4%	19.4%	21.9%	24.0%	1.10 (0.99–1.22) p = 0.08
Traditional/safe period/other	4.2%	6.6%	7.4%	9.7%	10.0%	1.44 (1.26–1.64) p<0.001
**Planning pregnancy in next three months**						0.62 (0.51–0.75) p<0.001
Yes	21.0%	14.0%	11.6%	7.4%	7.0%	
**Partners in past three months**						1.72 (1.54–1.92) p<0.001
Two or more	19.6%	8.9%	7.5%	6.1%	5.8%	
**Condom use with regular partner**						1.06 (0.95–1.17) p = 0.32
Always	10.2%	12.8%	12.3%	12.1%	10.7%	
**Condom use with other partners**						1.57 (1.28–1.93) p<0.001
Always	34.3%	50.0%	62.7%	62.8%	57.4%	
**Cleaned inside vagina in past three months**						1.25 (1.16–1.34) p<0.001
Yes	73.7%	85.2%	88.1%	85.6%	83.6%	
**Chance of catching HIV**						0.86 (0.79–0.94) p = 0.001
Likely	89.5%	87.9%	84.6%	87.7%	83.7%	

**1.** For each variable, we modelled the change in prevalence between visits using a random-effects logistic regression model adjusting for visit as a continuous variable and including a random intercept. This modelling approach adjusted for the expected additional correlation between multiple responses by the same participant. We calculated formal tests of significance using likelihood ratio tests, and the p-values from these tests are presented in the final column of the table.

**2.** *T. vaginalis* sample N = 220. Measurements made at recruitment (visit 1) and at 12 months (visit 5).

**3.** Pregnancy status was an initial study exclusion criterion. Prevalence at Visit 01 was therefore zero among eligible participants.

### Sexual behaviour, condom use and contraception

The proportion of women reporting two or more sexual partners at each clinical follow-up visit was significantly less than at baseline (p<0.001), whilst consistent condom use with non-regular partners was significantly higher at each time point (p<0.001; Table 4). There were no significant changes observed in reported condom use with regular partner. The prevalence of reported vaginal cleansing was significantly higher during follow-up than at baseline (p<0.001). A significant reduction was seen in the proportion of women who perceived themselves to be at high risk of HIV acquisition (p = 0.001).

Among the 1573 women attending a first clinic visit, anal sex in the previous three-months was reported by ≤1% at baseline, 6 and 12-months (data not shown).

Reported intent to become pregnant was significantly lower at each follow-up visit than at baseline (p<0.001) with a significant increase also observed in contraceptive prevalence (all methods combined) (p<0.001). However, there was no significant change in the proportion of women using highly effective contraceptive methods (pills/injection/IUCD) (p = 0.08) and the proportion of women with positive urine pregnancy tests at baseline, 6 and 12-months were similar (9.7, 10.0 and 9.3% respectively).

### Cohort retention

Among the 771 eligible women who attended at three-months, older women, those working in pombe shops or as *mamalishe*, those reporting fewer sexual partners in the last three months and women who were less mobile in terms of recent changes in accommodation and place of work had a significantly greater odds of re-attendance (Table 5). The adjusted odds of re-attendance were five times higher in women aged ≥35years than those aged <20years. Marital status, ethnicity, education level and literacy were not associated with re-attendance (data not shown).

**Table 5:** Factors associated with re-attendance^1^ among 771 eligible women attending at three months.

Characteristic	Total Number	% complete attendance	OR (95% CI) 2	Adjusted OR (95% CI) 3
**Age (years)**	n = 770		P<0.001	<0.001
<20	41	39.0	1	1
20–24	164	53.1	1.85 (0.98–3.49)	1.93 (1.01–3.70)
25–34	336	65.5	3.25 (1.77–5.96)	3.20 (1.71–5.99)
35+	229	81.2	7.38 (3.83–14.24)	5.40 (2.68–10.90)
**Facility type**	n = 771		P = 0.005	P = 0.19
Pombe*/Mamalishe*	517	71.8	1	1
Guesthouse/Hotel	115	53.0	0.55 (0.37–0.83)	0.65 (0.41–1.02)
Bar/Restaurant/Grocery	139	55.4	0.64 (0.44–0.94)	0.80 (0.52–1.23)
**Partners in past 3 months** 4	n = 766		P = 0.01	P = 0.08
None	124	74.2	1	1
One	505	67.5	0.81 (0.52–1.28)	0.75 (0.47–1.20)
Two	98	54.1	0.60 (0.34–1.06)	0.62 (0.34–1.12)
Three or more	39	46.2	0.45 (0.22–0.91)	0.56 (0.26–1.19)
**Condom usage**	n = 771		P = 0.05	P = 0.73
No partner	130	73.9	1	1
Never	466	68.5	0.88 (0.56–1.38)	1.00 (0.57 1.77)
Sometimes	116	53.5	0.58 (0.34–0.99)	0.94 (0.44–2.01)
Always	53	50.9	0.53 (0.28–1.01)	0.72 (0.34–1.54)
**Travel** 4, 5	n = 770		P = 0.01	P = 0.03
Low	527	69.6	1	1
Moderate	208	58.2	0.69 (0.50–0.96)	0.73 (0.51–1.03)
High	35	57.1	0.54 (0.27–1.08)	0.54 (0.27–1.09)
**Permanence** 4, 6	n = 771		P = 0.002	P = 0.01
High	342	75.2	1	1
Moderate	206	64.1	0.73 (0.50–1.08)	0.71 (0.47–1.06)
Low	221	53.4	0.56 (0.39–0.81)	0.59 (0.40–0.87)

**1.** Attendance was defined on a four–level [0, 1, 2 and 3] ordinal scale as the number of visits made after a second visit at 3 mo (i.e. zero, one, two, or three additional visits) and modelled using ordinal logistic regression. The OR is the estimated odds ratio of ≥k visits vs <k visits, assumed equal for all k (1,2,3). Complete attendance was defined as attending all three possible visits after a second visit at 3-months.

**2.** Odds Ratio adjusted for age.

**3.** Odds Ratio adjusted for age, clinic site, facility type, partners in past 3 months, travel, and permanence.

**4.** Test for trend used to assess significance of term in ordinal logistic regression.

**5.** Travel in the three months preceding the first clinic visit was categorised as: Low (zero nights away from home), Moderate (≥1 night but less than one continuous week away from home) and High (≥1 continuous week away from home on one or more occasions).

**6.** Permanence of home and workplace location was categorised as: High (lived in the same house and worked at the same facility during the past year); Moderate (lived or worked at the same location for <1 year and moved house no more than once in the previous year); Low (lived or worked at the same location for <1 year and moved house twice or more in the previous year).

## Discussion

Researchers in a variety of settings have conducted preparedness or feasibility studies in order to assess the suitability of potential study populations for future HIV prevention trials[5], [6], [7], [8], [10], [11], [12], [13]. Feasibility is critically dependant on a number of key epidemiological factors[1], [2]. HIV incidence rates need to be sufficiently high to support large scale clinical trials; cost-effective strategies for recruiting and retaining sufficient numbers of subjects need to be available or developed; the impact of effective risk reduction counseling and syndromic STI management on HIV and STI rates during follow up need to be taken into account; the ability to collect reliable, valid sexual behaviour data assessed; and key risks to future clinical trials, such as high pregnancy rates and differential losses to follow-up of those most at risk of HIV seroconversion, appraised and corrective strategies developed. There are also additional issues specific to vaginal microbicide trials that need to be taken into account such as product acceptability (among participants and their sexual partners)[39], [40], [41], [42], [43] and intravaginal practices[44], [45].

During this microbicide trial feasibility study in northern Tanzania, we were able to recruit and retain a large number of participants from a high-risk occupational cohort of women known to engage in transactional sex. Of the 2494 potential participants identified during community mapping, 1573 (63.1%) attended a first clinic visit and were enrolled directly into the feasibility cohort. Among a sub-cohort of 1020 HIV negative non-pregnant women attending a first visit who would have been considered broadly eligible to enroll in a microbicide trial, there were significant early losses to follow-up. Subsequent retention among a sub-cohort of 771 eligible women who attended a second clinic visit was however more satisfactory. Comparable retention rates have been reported in similar high-risk occupational groups and in female sex worker cohorts elsewhere in Africa and in SE Asia[46], [47], [48], [49], [50], illustrating the considerable logistical difficulties that need to be addressed when working with such study populations. Key factors likely to have contributed to the early losses observed in Mwanza include single clinic attendance by women who wanted to receive STI treatment alone but who had no intention of remaining in the study; attendance by women curious to know more about the study having attended community mobilization activities but who subsequently decided not to participate further; and difficulties tracing women in the community to remind them to re-attend clinic due to inaccuracies in baseline locator information and high cohort mobility[26], [37]. Many of these issues could have been addressed during a preliminary screening visit to identify and exclude women who are unlikely to re-attend, through improved participant tracing procedures and targeted community-based mobilization and liaison activities, all of which were subsequently successfully incorporated into the design of the MDP301 microbicide trial in Mwanza where 80–85% re-attendance figures were observed at each of the scheduled 3-monthly clinical follow-up visits.

The incidence of HIV observed during the feasibility study was lower than in a similar occupational cohort in Mbeya, Tanzania[48] or among female sex workers in Kenya, South Africa, Thailand, Benin and Cote d'Ivoire[47], [51], [52], [53], but higher than that observed among hotel, bar workers and women attending family planning clinics in the HPTN055 study in Moshi, Tanzania (HIV incidence 1.3/100PYs [95%CI:0.3–3.8]) [50]. It is difficult however, to define a suitable cut off level for HIV incidence above which prevention trials are likely to be feasible because in any given at-risk study population, feasibility depends on additional interrelated factors, principally the total sample size likely to be achievable and the risk of significant losses to follow up. For example, all of the HIV seroconversions observed in the Moshi HPTN055 study (N = 240) occurred among hotel and bar workers, who were also significantly less likely to re-attend at 12-months compared to women recruited from family planning clinics (81% vs. 93%; p<0.01)[50]. Prior to the MDP301 trial in Mwanza, a second, more intensive, participatory community mapping survey was conducted in May 2004, which identified a total of 3581 women working in 1106 facilities. We estimated that around 30–35% of these potential participants would be ineligible for trial entry based on HIV and pregnancy prevalences at first visit as described above and that an additional 20–25% would likely be unable to enroll for other reasons such as being unwilling to participate having attended a community-based mobilization meeting; unable to provide informed consent; or unwilling to receive their HIV test result prior to randomization. In addition, there are considerable challenges in attempting to contact all potential participants in this setting due to a variety of external contextual factors (e.g. not at work because child sick; sleeping following evening/night shift when fieldworkers visit the facility). The total sample size considered achievable was hence estimated at around 1300–1400 subjects. This essentially makes single-centre HIV prevention trials unfeasible in this setting, particularly when current candidate vaginal microbicides and HIV vaccines are expected to be only partly effective. For example, to detect a 50% effect size with 90% power at the 5% significance level in a two-arm trial, assuming an HIV incidence of 2.0/100PYs in the placebo arm as observed in potentially eligible women in the feasibility study, would require a total of 6300PYs of follow-up, not realistically achievable in this study population A more appropriate design was adopted in the MDP301 trial, in which women were recruited from six sites in four African countries, the target for subject recruitment in Mwanza was 1150 and the individual period of follow-up for the primary efficacy outcome was 12-months. In addition, given the modest overall HIV incidence observed in the Mwanza feasibility study cohort but the relatively high incidence seen among younger women working in modern bars and restaurants (4.1/100PYs [95%CI: 1.7–9.7]; Table 3), recruitment strategies to the MDP301 trial in Mwanza focused on women in this sub-population who appeared to be at greatest risk of HIV sero-conversion.

There was some evidence of differential loss to follow up during the feasibility study: younger, more mobile, arguably ‘higher-risk’ women were less likely to re-attend. In addition there were significant reductions in reported numbers of sexual partners and the prevalence of laboratory-confirmed STIs and vaginal infections observed and an increase in reported condom use with non-regular partners. An alternative explanation is that these findings are the result of effective clinical services for STI management and risk reduction counselling, as reported in similar studies elsewhere[48], [53]. Social desirability bias may also have played some role in modifying reported sexual behaviour over the period of follow-up. Whatever the explanations, our findings suggest that there may be a tension between recruiting ‘higher-risk’ women with higher HIV incidence to reduce sample size requirements, and the need to maintain high rates of follow-up.

Pregnancy rates were relatively high during the feasibility study but lower than those observed in some other at-risk cohorts, for example, among female sex workers in Madagascar, where an incidence of 53 pregnancies per 100PYs has been reported[43]. Low contraceptive prevalence and the lack of predictive value of stated intent to become pregnant are also cause for concern and represent a major risk to the successful conduct and completion of any future microbicide trial in this setting. In order to minimise this risk during the MDP301 trial, potential participants were advised in community meetings and during informed consent procedures that they should only consider enrolment if they were willing to forgo further pregnancies during the trial; that they should not enrol if they were considering pregnancy in the next 6–12 months; and that they should consider using highly effective family planning methods such as long-acting intramuscular progesterone, oral contraceptive pill or the intra-uterine contraceptive device (IUCD). These strategies were partly successful: in the first 18-months of the main phase III trial in Mwanza, the incidence of pregnancy was 12.3/100PYs (95%CI:8.9–17.0), far lower than that observed during this feasibility study.

In addition to the epidemiological considerations above there are a range of other factors that contribute to feasibility, principally ethical considerations; a full discussion of which is beyond the scope of this paper but which are described in detail elsewhere[26]. The prevalence and nature of intravaginal practices are also important since internal cleansing and the use of vaginal inserts such as herb poultices could have a significant impact on the apparent efficacy of a future trial product, particularly if these are carried out immediately before or after sex when they may inactivate or remove the candidate microbicide under investigation. In Mwanza, detailed quantitative and qualitative research was conducted during the feasibility study in order to better understand the nature, timing and different types of intravaginal practices prevalent in this setting (*Allen C et al, submitted, October 2009*).

The Mwanza feasibility study data suggest that women working in food and recreational facilities in Tanzania and other parts of sub-Saharan Africa are likely to be suitable study populations for microbicide and other HIV prevention trials. Effective locally-appropriate strategies to address high pregnancy rates and early losses to follow-up are essential to minimise risk to clinical trials in these settings.
